# Vertical Geochemical Variations and Speciation Studies of As, Fe, Mn, Zn, and Cu in the Sediments of the Central Gangetic Basin: Sequential Extraction and Statistical Approach

**DOI:** 10.3390/ijerph15020183

**Published:** 2018-01-23

**Authors:** Manoj Kumar, AL. Ramanathan

**Affiliations:** 1 Department of Environmental Science, School of Earth, Environment & Space Studies, Central University of Haryana, Jant Pali, Mahendergarh 123029, India; 2 School of Environmental Sciences, Jawaharlal Nehru University, New Delhi 110067, India; alrjnu@gmail.com

**Keywords:** elemental contamination, enrichment, sequential extractions, residual fraction, oxidizable fraction, color code, mineralogy

## Abstract

A geochemical and speciation study of As, Fe, Mn, Zn, and Cu was performed using sequential extraction and statistical approaches in the core sediments taken at two locations—Rigni Chhapra and Chaube Chhapra—of the central Gangetic basin (India). A gradual increase in the grain size (varying from clay to coarse sands) was observed in both the core profiles up to 30.5 m depth. The concentrations of analyzed elements ranged as follows: 6.9–14.2 mg/kg for As, 13,849–31,088 mg/kg for Fe, 267–711 mg/kg for Mn, 45–164 mg/kg for Cu for Rigni Chhapra while for Chaube Chhapra the range was 7.5–13.2 mg/kg for As, 10,936–37,052 mg/kg for Fe, 267–1052 mg/kg for Mn, 60–198 mg/kg for Zn and 60–108 mg/kg for Cu. Significant amounts (53–95%) of all the fractionated elemental concentrations were bound within the crystal structure of the minerals as a residual fraction. The reducible fraction was the second most dominant fraction for As (7% and 8%), Fe (3%), Mn (20% and 26%), and Cu (7% and 6%) respectively for both the cores. It may be released when aquifers subjected to changing redox conditions. The acid soluble fraction was of most interest because it could quickly mobilize into the water system which formed the third most dominating among all three fractions. Four color code of sediments showed an association with total As concentration and did not show a relation with any fraction of all elements analyzed. The core sediment was observed enriched with As and other elements (Cu, Fe, Mn, and Zn). However, it fell under uncontaminated to moderately contaminate which might exhibit a low risk in prevailing natural conditions. X-ray diffraction analyses indicated the availability of siderite and magnetite minerals in the core sediments in a section of dark grey with micaceous medium sand with organic matter (black).

## 1. Introduction

Over the past four decades, sequential extraction procedures have accomplished a rapid increase in their application [1]. The emergence of sequential extraction was primarily focused on elucidating potential impacts of sediment-bound elements triggering toxicity to water quality [1]. However, its primary application was to measure the soil quality and toxicity level as well [2,3,4]. By the early 1990s, some studies were using the scheme to fractionate the bound elements to estimate the potential toxicity in various substrates [1]. Sequential chemical extraction is also termed as “operational speciation” in which the reagent used to extract the sample to define the species [1]. Sequential extraction is thus now widely accepted and adopted to assess potential mobility and risks (hence, potential bioavailability and toxicity) of toxic elements in the environment [1]. Although this process is time-consuming, it offers the advantage of providing information about the origin, mode of occurrence, physicochemical availability, and transport/cycling of elements [4,5].

Sequential extractions give semi-quantitative information on the elemental variability of operationally defined geochemical fractions in soils, sediments and other solid substances [6]. Sequential extractions can indicate the pools or sinks of elements that are potentially available under challenging conditions [6]. The chemical and biochemical interactions between water and the geological materials trigger the easily labile fraction to enter into the solute, hence can increase the toxicity level [7]. The toxicity of elements vary with their chemical forms present in the system rather than their total elemental contents, and therefore, speciation studies gradually gain importance [5,8]. Moreover, as an estimate, the total concentrations, the mobility, and availability of As and other potentially toxic elements must be determined to explain their behavior in soils and to check possible toxic hazards [9]. The importance of elemental mobility and bioavailability in the environmental risk assessment of contaminated soils is widely recognized [5,10]. Mobility and bioavailability of elements in soil depend on the speciation of the element rather than the total concentrations [10].

The Community Bureau of Reference (BCR) method has been widely used in the fractionation of various elements, including As, for soil and sediment and other substances [11]. The determination of the chemical forms of the selected elements was carried out by the three-step sequential extraction, in accordance with the modified BCR procedure, which enables the estimations of three main fractions and an additional residual fraction [12]. The viewpoint after the sequential procedure was that each successive reagent would dissolve different components so from the study of fractionation pattern of the elements provide concluding information about the mobility, pathways or bioavailability of the element or an assessment regarding the potential hazards [12].

Various studies from the lower Gangetic basin [13,14,15,16,17] and a very few in the central Gangetic basin [8] have attempted to identify an association of aquifer sediment color with the level of As in groundwater. These studies revealed that Holocene grey sand aquifers exhibit elevated concentrations of dissolved As > 10 μg/L, the WHO (2011) guideline and BIS (2012) standard for safe drinking water, whereas brown sediment aquifers may be free from As pollution [13]. A recent study has also been reported that As concentration may show higher levels if the grey sands are mixed with organic matter [8]. Sediment extraction from a study reported low As concentration below 2.5 mg/kg in oxidized sediments [18]. This concept was used by von Bromssen et al. [15] to develop a four color (black, white, off-white and red) hypothesis by incorporating the color perception of the local drillers. The color code symbolizes that highest risk associate with black color sediment and gradually reduces toward red [17]. The color pattern also tells about the redox status of the aquifer and in general black sediment indicates the reduced condition of the aquifer, and it decreased gradually towards brown color sediments. Availability of ions that compete with As for adsorption sites (e.g., PO_4_^3−^, and Si) may also trigger the release of sediment-bound As [18].

The occurrence of elevated concentrations of As in the central Gangetic basin has been proven by various studies that have examined water, subsurface sediments and agricultural soils [8,19,20,21,22,23,24]. All these studies focused on total elemental estimation except for a few that concentrate on speciation studies in sediments [21]. Very few efforts have been directed to speciation studies on As and other elements like Cu, Zn, Mn, Fe, and other elements in the subsurface sediment and its association with the sediment color in the central Gangetic basin; so it is crucial to fill this gap. The present study was conducted by taking key objectives as to document the bioavailability and environmental hazards associated with operationally defined chemical forms of As and other elements (Cu, Fe, Mn, and Zn) using the speciation technique. An attempt also has been directed to understand the role of mineralogy in As and other elemental occurrence and mobilization in aquifers, and finally to check the applicability of the four color code for sediments in As speciation occurrence in the central Gangetic basin of India.

## 2. Materials and Methods

### 2.1. Site Area Description

**Figure 1:**
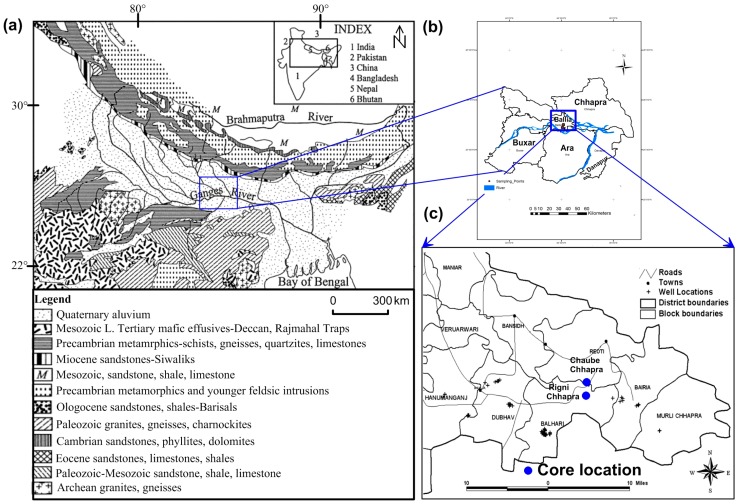
Study area map (**a**) Geological map of the Ganges and Brahmaputra drainage basins (modified from [28]; (**b**) surrounding area near the study location (**c**) Ballia district (study area) marked with core locations (modified from [25]).

The present research was carried out in Ballia district, Uttar Pradesh, at two locations namely Rigni Chhapra and Chaube Chhapra, sited on the bank of the River Ganges (Figure 1). Ballia district is the easternmost part of Uttar Pradesh, India, covering a total area of 3168 km^2^ with a total population of 2.75 million, and lying between 25°33′ to 26°11′ N latitudes and 83°38′ to 84°39′ E longitudes. Rigni Chhapra and Chaube Chhapra villages in the Ballia district were chosen for drilling studies based on the As contamination observed during field survey using a portable test kit (results are not provided in the current study). This region is highly affected by As poisoning in subsurface water, soils and sediments [22,25] and known for elevated As concentration distribution in patches in the state’s groundwater supplies [26]. The contribution of surface water irrigation is only 27.4% while remaining 72.6% extract from the ground [27].

### 2.2. Geomorphology and Hydrogeology of the Study Area

Ballia district forms an interfluves zone and builds a plain topography which is flood-prone being located in the interfluves region of two perennial rivers: the Ganges in the north and the Ghagra in the south (Figure 1a). The significant parts of the Gangetic basin consist of the interfluves upland terrace surface, which represents the upper unit of older alluvium. River terrace surface and active floodplain areas are characterized by newer alluvium (Holocene) deposits which are mainly form reduced or the unoxidized subsurface environment. It consists of organic-rich sediment, silt and clay deposited in low-lying fluvial and fluviolacustrine setting (*Surha Tal*, *Reoti Tal* and *Sikandarpur Tal* and other abandoned channels in the form of *Tal* and ponds), derived from the Himalayas and Peninsular Craton [29]. It forms the topmost few meters of a thick layer of the newer alluvium which covers all surfaces. Newer alluvium consists of weathered material brought from the Himalayas via an entrenched channel having undergone further chemical weathering in reducing conditions promoting the mobilization of As [29]. A study based on mineralogical and stratigraphic data indicates that the river Ganges changed its course several times during the Holocene [28].

Various studies already have documented the geography and geomorphology of the study area [8,29,30,31]. It is a repository of Quaternary deposits of the Ganges basin and categorized into three major setups as (T_0_), (T_1_) and (T_2_) known as active floodplain, river valley terrace surface and upland interfluves surface respectively [29]. These surfaces are characterized by the mare depositional surfaces, which made a thick formation of sediments of Holocene over Pleistocene. The Ganges foreland basin is a consequence of sediments weathered from the Himalayas and a contribution from Peninsular Craton [29,30]. Aquifers of Holocene sandy sediments are unconfined or semi-confined in the central Gangetic basin which is the main source to meet the water requirements to the residing population. Most of the tubewells extend to shallow aquifers <50 m below ground level (mbgl).

### 2.3. Sample Collection and Preparation

Sediment core collection was carried out with the help of local resources and using a technique (hand flapper drilling method) to a depth of 30.5 m at Rigni Chhapra and Chaube Chhapra, respectively. Samples (*n* = 24) were collected—two samples of surface soil and others at depths of 1.5, 3, 4.6, 6.1, 9.1, 12.2, 15.2, 18.3, 21.3, 24.4 and 30.5 mbgl at both the locations. A small pond was dug and it was always kept full of water. Drilling followed the principle of suction in which sediment pieces were thrown up along with water. The selection of the samples was based on the grain size and different color of sediment. These selected samples were carefully wrapped in black plastic bags and stored in an ice box and then transported to the laboratory in School of Environmental Sciences (SES), Jawaharlal Nehru University (JNU), New Delhi. All the samples were freeze-dried in the laboratory. Finally, samples were ground into a powdered form with agate and mortar and stored for further processing. Samples for grain size analysis were stored separately.

### 2.4. Chemical and Reagents

Milli-Q water (Milli-Q Plus system; Millipore, France) with 18.2 MΩ·cm at 25 °C resistivity was used throughout the experiment and other dilution purposes. Analytical grade reagents were used in the digestion and extraction processes. Concentrated nitric acid (HNO_3_) and 30% hydrogen peroxide (H_2_O_2_) were obtained from Fisher Chemicals, India. All stock solutions were kept in a state of darkness in a cold room at 4 °C. Standards were prepared freshly from their respective stock solution on the same day of the analysis.

### 2.5. Sediment Sample Digestion and Sequential Extraction Procedure

Digestion of core sediment samples was performed using a method developed by Shapiro [32]. Ground sediment samples (approximately 0.10 g of each) were transferred directly into Teflon crucibles and aqua regia (HNO_3_ and HCl ratio 3:1, 2 mL) and HF (5 mL) were added to each crucible. Samples were allowed to predigest by standing open for 30 min before sealing the vessels. After that, the vessel was sealed carefully. The crucibles were heated for 1 h at 100 °C in an oven and allowed to cool down at room temperature. Each sample was visually tested to clear solution indicating the total digestion (no residual) of the sediment. An amount of (5.6 g) boric acid crystal (H_3_BO_3_) was dissolved in distilled water (2 mL) and then added to bomb content up to 100 mL. The solution was transferred to polypropylene bottles. The sample was left undisturbed overnight to allow the formation and settling of borosilicate from the solution. 0.45 µm Millipore filter paper was used to separate the gelatinous precipitate from the solution for elemental analysis. A three-step sequential extraction procedure namely modified BCR scheme was used to target chemical forms [11]. The details of the extraction method have been shown in Table S1 in the Supplementary Materials. In the table, step 1 represents the acid-soluble fraction, which comprises exchangeable and carbonate-bound fraction was obtained by applying (0.11 M, acetic acid; pH, 7); step 2 represents being bound to Fe/Mn oxides was obtained using (0.5 M, hydroxylammonium hydrochloride; pH 1.5–2); step 3 represents being bound to organic material and sulphides was extracted by applying (8.8 M, hydrogen peroxide; pH 2–3, 1 M, ammonium acetate; pH 2); and an additional residual fraction was obtained after digestion with aqua regia.

### 2.6. Instrumentation and Analysis

Atomic absorption spectroscopy (AAS, M Series, Thermo Scientific, Cambridge, UK) was used for the estimation of the elements, i.e., Cu, Fe, Mn, Zn, and As. The estimation of Fe, Mn, Zn, and Cu was performed using the flame method while for As, hydride generation (HG-AAS, <1 μg/L detection limit in solution matrix with ±5% precision) on AAS was used. The concentration of As and other elements in sediments was estimated on the solute basis, and it was further calculated on a dry weight basis. Laser diffraction particle size analyzer (S3500, Microtrac, Pennsylvania, PA, USA, coupled with a Microtrac SDC unit) was used to perform the grain size distribution analysis. Analysis work was done in the laboratory of the SES, JNU, New Delhi, India. pH was measured using a portable multiparameter meter (HACH-Sension 156, Hach Company, Loveland, CO, USA). Silica, phosphorus and aluminum were extracted by adopting the Shapiro method [32]. Organic matter and carbonate content were determined in accordance the loss on ignition procedure [33,34]. The estimation of organic carbon estimation was done using the Walkley Black Method [35]. Estimation of total carbon and nitrogen was done using a CHNSO Elemental Analyser (Euro EA, Pavia, Italy). The X-ray Diffraction (XRD) analysis (powder) was done by using the X’pert PRO (PANalytical, EA, Almelo, The Netherlands) at the JNU advanced instrumentation research facility. Detection limit and analytical precision of the instrument (AAS) for all elemental analysis was calculated as:

Detection limit =3×Standard deviation of blanks

$$\mathrm{Analytical}\;\mathrm{precision}\;\left(\%\right)\;=\frac{\mathrm{the}\;\mathrm{standard}\;\mathrm{deviation}\;\mathrm{of}\;\mathrm{standards}}{\mathrm{means}\;\mathrm{of}\;\mathrm{standards}}\times 100$$

The instrumental detection limits for Fe, Mn, Zn and Cu were 0.02, 0.01, 0.01 and 0.03 mg/L in the solution matrix, respectively.

### 2.7. Quality Assurance and Quality Control of the Analysis

Certified reference material (CRM’s) BCR 320 river sediments (*n* = 5) was used to verify the results of As and other elements in sediments. The certified values of As, Mn, Zn, Cu were 76 ± 3.4, 0.8, 142 ± 3, 44.1 ± 1 mg/kg respectively; while for the observed values detected in this study were 73.3 ± 2.2, 0.76 ± 0.03, 137.2 ± 3.7, 42.1 ± 2.3 respectively. For speciation of As and other elements, an internal check was performed by comparing the results of the total digestion to the total amount of element extracted by different reagents during the sequential extraction procedure. The recovery of the sequential extraction method was calculated using the given equation:

$$\mathrm{Recovery}\;\left(\%\right)=\;\frac{C_{\mathrm{acid}\;\mathrm{soluble}}+C_{\mathrm{reducible}}+C_{\mathrm{oxidizable}}+C_{\mathrm{residual}}}{C_{\mathrm{total}\;\mathrm{digestion}}}\times 100$$

The sums of the four fractions were found in good agreement with the total digestion results, with satisfactory recoveries in case of As (91–99%), Fe (91–94%), Mn (96–99%), Zn (90–94%) and Cu (91–94%). It was observed that all the extraction steps showed little under-extraction even in case of CRM’s.

### 2.8. Statistical Work

A Pearson correlation matrix was performed with the help of IBM SPSS Statistics 19 (IBM Corp., New York, NY, USA). A *p* value of <0.01 was considered to indicate statistical significance.

#### 2.8.1. Risk Assessment Code (RAC)

The risk assessment code (RAC) was performed on the results of the speciation data. Table S2 in the Supplementary Materials describe the criteria and indicates the percentage of the fraction which can be released as easily exchangeable and carbonate fractions (i.e., acid-soluble fraction). It may vary from <1% of the total elemental concentration which is an acid-soluble fraction (considered safe) for the environment while sediment releasing >50% of the total elemental concentration in the same fraction has to be considered (highly dangerous) and can readily enter the food chain [36].

#### 2.8.2. Geo-Accumulation Index (I_geo_)

The Geo-accumulation Index (I_geo_), proposed by Muller [37], is often used for quantitative assessment of the elemental pollution situation in the water-sediment environment. Igeo can be used to know the variation of elemental distribution as well as the influence of human activities on the environment [38]. Geo-accumulation Index is expressed as:

$$I_{\mathrm{geo}}=\log_{2}(\frac{\mathrm{Cn}}{1.5\mathrm{Bn}})$$

where Cn = Content of the element in the sample; Bn = Background content of the element in the deposit which is taken from the “average shale” provided in reference [39]. Factor 1.5 is a constant, for considering rock differences, diagenesis and other factors that may cause fluctuations in the background content. According to the magnitude of the geo-accumulation index, the pollution degree of the element could be divided into seven grades (0−6) as shown in Table S3 in the Supplementary Materials. The highest grade (I_geo_ = 6) represents 100 fold accumulation above background concentration.

#### 2.8.3. Enrichment Factor (EF)

Enrichment factor is based on the standardization of the tested element in the sample with a reference. The use of the EF for the assessment of sediment contamination with elements has been suggested by Baut-Menard and Chesselet [40]. The most common reference elements are Sc, Al, Mn and Fe [41]. Aluminum was used as a conservative element in this study since it was the most accurate and the most precisely measured major element, and it has been the most commonly used normalizing element in the geochemical literature [42]. The equation for the EF expressed:

$$\mathrm{EF}=\frac{\left(\frac{M}{\mathrm{Al}}\right)\mathrm{sediments}}{\left(\frac{M}{\mathrm{Al}}\right)\mathrm{Shale}}$$

where M/Al is the ratio of the concentration of element M to Al. M = concentration of the element examined Al = level of Aluminum in sediment and shale respectively. It has been categorized into five categories which may vary from deficiency to minimal enrichment (EF < 2) to extremely high enrichment (EF > 40) as shown in Table S4 in the supplementary material.

## 3. Results and Discussion

### 3.1. The Grain Size of the Core Sediments and Lithographs Reconstructions

**Figure 2:**
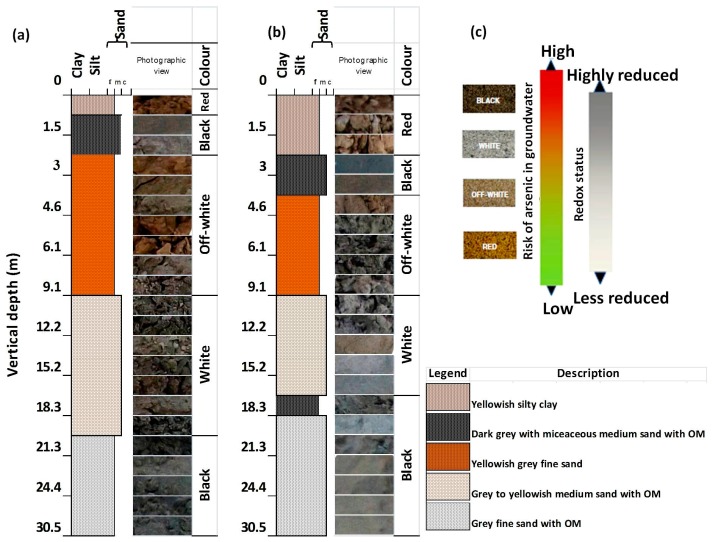
Lithologs prepared from core profile samples along with their corresponding fresh sediments photographic view (**a**) Rigni Chhapra (**b**) Chaube Chhapra (**c**) Four color sands with corresponding risks of As concentration in groundwater under varying redox status adopted from reference [17].

Litholog prepared from the collected core samples characterized sandy channel-fill formation with silty over bank deposit at the top (Figure 2a,b) indicating a fluvial environment of deposition [43]. Sand was the dominant fraction in overall lengths of both the cores. In upper layers, silt and clay percentage were approximately 80% at Rigni Chhapra and 90% at Chaube Chhapra, and it was decreased with the depth vertically (Table 1). At some layers, there was decidedly less or no clay fraction observed. There were many fluctuations in size fractions with depth. Soil texture has held on soil water retention characteristics, leaching and erosion potential, organic matter dynamics and carbon sequestration [44]. The sediments were categorized into five distinct lithofacies including yellowish silty clay, dark grey with micaceous medium sand, yellowish grey fine sand, grey to yellowish medium sand, grey fine sand accordance with the texture and lithology for both cores. The logic behind it was to make a direct and consistent comparison of sediment color in the central Gangetic basin. Fresh sediment photographic view also has been shown along with the lithofacies (Figure 2a,b). A four-color tool (Figure 2c) was served to categorize the samples into four colors. It was derived from modified Munsell Colour Chart by a comparative analysis of 2240 sediment samples [17]. An evident variation in color, i.e., red, black, off-white, white, black was observed from top to bottom in both the cores sediments. Thin clay lenses were found in both the cores at a depth of 1.5 and 3 m at Rigni Chhapra and Chaube Chhapra respectively. Another clay lens was also observed in the core of Chaube Chhapra at a depth of 18 m.

### 3.2. Physical Characteristics of the Vertically Subsurface Sediments

**Figure 3:**
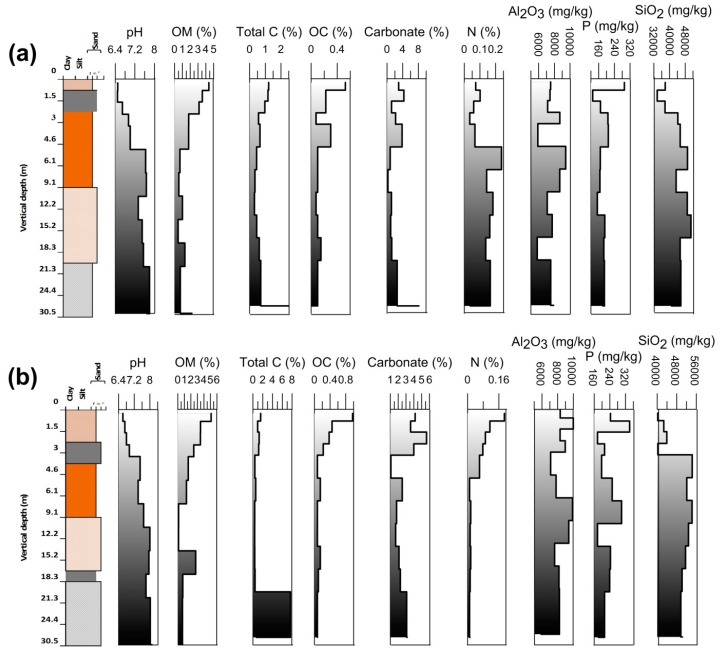
Physical parameters (pH, organic matter, total carbon, organic carbon, carbonate, N, Al_2_O_3_, P, and SiO_2_) of core sediments (**a**) Rigni Chhapra (**b**) Chaube Chhapra.

Core sediment samples were analyzed for various physical parameters, i.e., pH, organic matter, total carbon, organic carbon, carbonate content, nitrogen, Al_2_O_3_, P, and SiO_2_ and presented in Table S5 in Supplementary Materials. All physical parameters of the sediment have been represented by comparing with the corresponding lihologs in (Figure 3a,b). The surface sediment was relatively acidic at both the places. Both the cores were collected from an agricultural land containing residues of the crops and other organic substances (e.g., humic acids) [45], which may attribute to decrease in the pH. Except at the surface, pH showed an increasing trend vertically in both the cores. There was an almost same trend in organic carbon and organic matter at both the locations indicating the same source for both the constituents. The average percentage of organic carbon in the sediments was observed to be 0.17 for Rigni Chhapra (maximum 0.53% in topsoil and minimum 0.02% at 30.5 mbgl) and 0.23 for Chaube Chhapra (maximum 0.98% in topsoil and 0.08% at 6.1 and 30.5 mbgl respectively). The average percentage of organic matter was 1.5 for Rigni Chhapra (maximum 4.39% in topsoil and minimum 0.46% at 18.3 mbgl) and 1.33 (maximum 5.1% in topsoil and minimum 0.11% at 15.2 mbgl) for Chaube Chhapra.

The presence of maximum organic matter in the topsoil is governed by agricultural practices and high microbial activity due to the availability of nutrients [46]. Organic matter content decreased continuously vertically below due to the lack of these conditions. There was another peak of organic matter at 30.5 mbgl in Rigni Chhapra due to the presence of high amount of clay content. There was decidedly less organic matter content in the section of 9 to 18.3 mbgl respectively, in both the locations due to the dominance of medium to coarse sands at that depth in both the core sediments. The average percentage of carbonate content was 2.54 for Rigni Chhapra (maximum 8.13 at 30.5 mbgl and minimum 0.22% at 12.2 mbgl) and 2.6 (maximum 5.63% at 3.0 mbgl) for Chaube Chhapra. The carbonate content was almost stable up to a depth of 25.9 mbgl. Further, there was a sudden increase in carbonate content in both the cores. In Rigni Chhapra there was maximum carbonate content (8.13% at 30.5 mbgl) indicating kankar (calcareous) deposition with a black color clay layer, while at Chaube Chhapra there was less carbonate than at Rigni Chhapra.

Nitrogen showed an increasing trend in the core of Rigni Chhapra, which was very close to the channel of the river Ganges. While at Chaube Chhapra it showed a decreasing trend. Al_2_O_3_ and P attained the same trend in both the cores due to very less difference in the lithology of both the locations and also indicating geogenic origin only. Silica content was lower in the upper layers but increased vertically due to the presence of medium to coarse sand.

### 3.3. Arsenic and Other Elements in Vertical Profile

**Figure 4:**
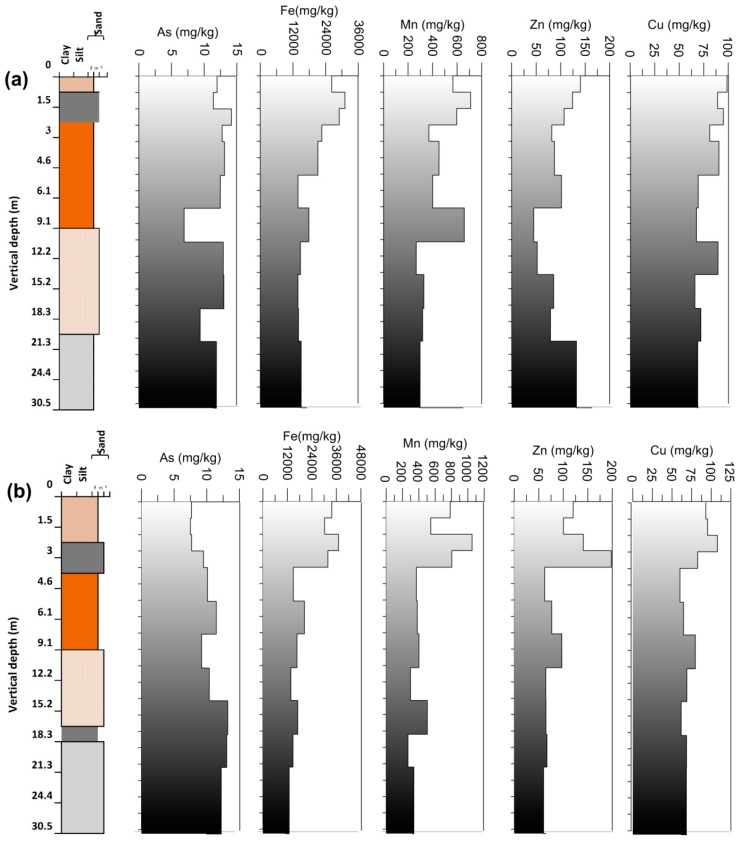
Vertical variability of As and other elements (Fe, Mn, Zn, and Cu) distribution in (**a**) Rigni Chhapra (**b**) Chaube Chhapra.

Vertical depth profiles of As concentration variation in both the cores are presented in (Figure 4a,b). The average concentration of As at Rigni Chhapra was 11.8 mg/kg (maximum 14.17 mg/kg at 3.0 mbgl and minimum 6.94 mg/kg at 12.2 mbgl) and at Chaube Chhapra it was reported as 10.2 mg/kg (maximum 13.21 mg/kg at 18.3 mbgl and minimum 7.5 mg/kg at 1.5 mbgl) as indicated in Table 2. It lacked any specific trend in As concentration with vertical depth. A more significant amount of As (14.17 mg/kg) was reported at a depth of 3 m followed by (13.1 mg/kg) at a depth of 6 m vertically below. These values exceeded the average crustal values of As (13 mg/kg). A dark grey micaceous sand and clay with organic matter was observed in this section. Clay can absorb As because of the oxide-like characters of their edge [47]. The total extracted concentration of As obtained from subsurface sediments was comparatively higher than the top layer sediments and showed a significant difference inferred the source of As as the subsurface sediment.

In the core sediments of Chaube Chhapra, the concentration of As was found comparatively lower (7.5–9.5 mg/kg) in the upper yellowish silty clay and yellowish grey fine sands which depicted red to off-white sediments color respectively. A higher concentration (10.11–13.21 mg/kg) was observed in lower sections of core profile with white to black color sediments. The trend was different for core profile of Rigni Chhapra whereas a higher As concentration was also observed in the upper section. It could be due to leaching from the topsoils in this area is periodically flooded throughout the year. However, a higher As concentration was also observed in white to black color sediments (lower section) in the core profile of Rigni Chhapra. The concentration of As varied with the color and grain size of the sediment in the Gangetic basin [43]. A lower As concentration was reported in brown to a yellowish color with coarser sediment while a grey to black color with fine grain size contained comparatively higher concentrations [36]. An excellent correlation (r^2^ = 0.96) was observed between As in soil and sediment samples in Chhattisgarh, situated in central India [48].

The average Fe concentration at Rigni Chhapra was 19713 mg/kg (range 13,849–31,088 mg/kg); minimum at 18.3 and maximum at 1.5 mbgl while at Rigni Chhapra the average Fe concentration was 21,183 mg/kg (range 10,936−37,052 mg/kg); minimum concentration was observed at depths of 30.5 and maximum at 3 mbgl respectively. A decreasing trend in the concentration of Fe was observed in both the cores which have an association with the clay formation containing organic matter and carbonate content. The difference between concentrations was meager comparatively at both the locations. An element like Fe forms an association with the sulfide/organic matter of the sediment at all depths [49]. Arsenic releases from reductive dissolution of Fe oxyhydroxide which exist in the aquifer as dispersed phases, such as a coating on the sedimentary grains. Microbial metabolism of the sedimentary organic matter is the driven force of Fe reduction in alluvial shallow aquifers [50].

The average concentration of Mn at Rigni Chhapra was 468.25 mg/kg (range 267–711 mg/kg); the minimum concentration was observed at 15.2 and maximum at 1.5 mbgl while for Chaube Chhapra the average concentration was observed 505.33 mg/kg (range 267–1052 mg/kg). The minimum concentration depicted at a depth of 21.3 m and maximum concentration at 3 mbgl (Table 2). The higher concentration of Mn at both the locations was up to 3 m depth and showed an association with the silty and clay formation below, where an irregular trend was observed.

Zinc is a chalcophile element having less sensitivity to redox processes. It forms distinct sulphide phases but fewer pyrite forms [51,52,53]. Average concentration of Zn at Rigni Chhapra was 100 mg/kg, maximum level (163.8 mg/kg at the depth 30.5 mbgl) and minimum concentration (45 mg/kg at 12.2 mbgl) respectively while the average Zn concentration at Chaube Chhapra was 92.97 mg/kg (range 59.8−198 mg/kg) having minimum concentration at 24.4 mbgl and maximum at 4.6 mbgl. The vertical trend in Zn concentration was comparative to the trends of Carbonate, Fe, and Mn. Zinc showed affinity with Fe and Mn in the suboxic environment and to sulphide and carbonate in an anoxic environment [51,54,55,56]. The affinity of Zn has also has been observed with organic matter [57].

The concentration of Cu in crustal rocks ranges from about 10 to a few hundred mg/kg with an average concentration of 70 mg/kg. It forms strong complexes with organic molecules which are typically the second strongest (after mercury) of any of the elements [57,58]. Copper has an affinity with sulfide minerals especially in anoxic environment and may associate with pyrite [59]. The average concentration of the Cu at Rigni Chhapra was observed as 79.8 mg/kg (maximum 98.8 mg/kg in topsoil and minimum 66 mg/k at 18.3 mbgl). At Chaube Chhapra Cu concentration was 76 mg/kg (maximum 107.9 mg/kg at 3 mbgl and minimum 60 mg/kg at 6.1 mbgl). The concentration of Cu exceeded the average crustal value (45 mg/kg) at both the locations having their significance in the aquifer geology. Higher concentration of Cu in the upper layers may be attributed to anthropogenic contribution and presence of high organic matter with clay particles, except in the upper layers where the concentration of Cu was within average crustal value and had the same trend at both the places. In case of all the elements analyzed, a higher concentration was observed in the upper sections of the formation. Clay zone recorded high elemental concentration in a seasonally waterlogging agricultural field in Eastern Ganges Basin [60]. The probable reason may be the characteristic of fine-grained sediments to absorb more moisture and it’s to tendency hold more organic matter as well as the elemental concentration via formation of chelates [49].

### 3.4. Arsenic and Other Elements (Cu, Fe, Mn, Zn) Speciation Studies in Vertical Sediments

**Figure 5:**
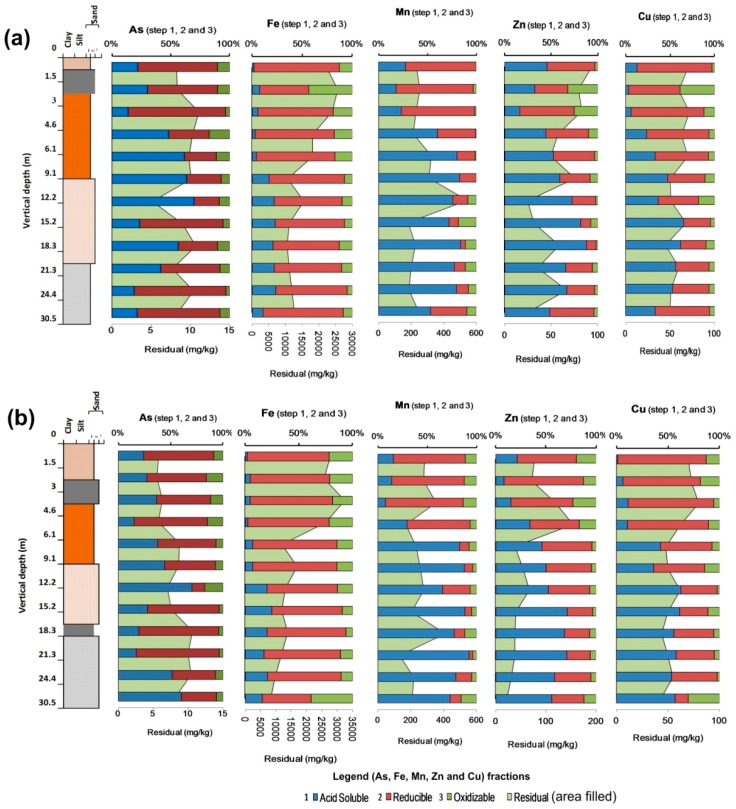
Percentage (leachable) and concentration of residual fractions (mg/kg) of As, Fe, Mn, Zn and Cu in each sequential step of the extraction procedure for sediment core profile of (**a**) Rigni Chhapra (**b**) Chaube Chhapra.

A modified BCR three-step sequential extraction procedure was adopted to get targeted chemical fractions from sediment profiles of two cores. Results are represented along with the litholog prepared from the sediment characteristics to make it convincing for the comparison (Figure 5a,b). Figures are representing the percentage of potentially leachable fractions and concentration of residual fractions (mg/kg) for elements, As, Fe, Mn, Zn and Cu in each step of the sequential extraction procedure for Rigni Chhapra and Chaube Chhapra. A bulk amount of all the analyzed elements were observed to be bound with the residual fraction suggesting its non-availability under prevailing natural conditions. Therefore a parallel plot was designed for labile/available fractions (i.e., acid soluble, reducible and oxidizable) in percentages excluding the residual which was presented on the secondary axis in mg/kg. The average pseudo-total elemental (total extraction) concentration in the core profile of Rigni Chhapra and Chaube Chhapra for As were observed as 11.8 and 10.2 mg/kg respectively, while the sum total concentrations were 10.3 and 9 mg/kg. A significant amount of As was bound to residual fraction 88% (9.1 mg/kg) and 88% (7.9 mg/kg) at Rigni Chhapra and Chaube Chhapra respectively which is non-available under prevailing natural conditions. Reducible fraction (bound to Fe/Mn oxides) was the second dominating fraction which bounded As to it except for the depth of 4.5 to 12 mbgl in Rigni Chhapra and at 12.2 mbgl in the core profile of Chaube Chhapra. Acid soluble (associated with exchangeable and carbonate bound) was the third dominating fraction followed by oxidizable (bound to the sulphides and organic matter). The corresponding percentage distribution of all three fraction for Rigni Chhapra observed as reducible, acid soluble and oxidizable were 61 (7% of the total extraction), 31 (4% of the total extraction) and 8 (1% of the total extraction), respectively. However, for the core profile of Chaube Chhapra, the percentage was 62 (8% of the total extraction), 29 (3% of total extraction) and 9 (1% of total extraction), respectively. The distribution of all these three fractions varied with different depths (Figure 5a,b).

The association of As with the reducible fraction as the second most dominant fraction suggests that it plays a vital role in its leaching into the environment. The percentage of the As fraction was higher in the upper section and bottom of the core profile where Fe and Mn concentration were also higher. The acid soluble fraction was higher in the middle section (highest at a depth of 12.2 mbgl) of the core profile of Rigni Chhapra while in the core profile of Chaube Chhapra. It was found to be dominated in the upper section (3 mbgl) and bottom sections (30.5 mbgl; the depth where most of the tubewell tapped were observed for groundwater extraction, although at these depths the carbonate content was not observed to be of a higher amount. These depths were, however below the water table (>6 mbgl), and it could be the outcomes of the leaching from the upper section containing higher carbonate content (Figure 3). A higher acid soluble and lower reducible As concentration was observed in the yellowish grey fine sand (4.5–12 mbgl) of an off-white color. It may be due to leaching of a potentially mobile faction of acid soluble fraction from the upper section. No clear association of any fraction was observed with the four color sand in both the core profiles. The As concentration bound to oxidizable fraction was least and associated with depths having fine sand containing organic matter.

For Fe, the fractions in both the core sediments followed a trend as residual (95%) > reducible (3) > oxidizable (1%) > acid soluble (1%)—in that order. The retention of a higher amount of Fe in the residual (non-available) fraction implied its nature bound in the crystalline iron peroxides (e.g., goethite, magnetite, haematite) [61]. Residual fraction also dominated for Fe and Zn (≈60%) in the sediments of lakes Qarun and Wadi El-Rayan, Egypt [5]. The second most dominating fraction of was Fe associated with the reducible fraction suggesting that it plays an important role in the leaching of elements into the environment. It was very high (70−71% of labile fractions) throughout the profile in both the core sediment among all the three labile phases (acid soluble, reducible and oxidizable) (Figure 5a,b). Acid soluble and oxidizable fractions were observed least in both the cores and exhibited an inverse relation vertically below, while the concentration of reducible fraction remained almost constant throughout the core profiles of both the locations. A low quantity of Fe in the oxidizable and acid soluble fractions implies low release and occurrence under suboxic and neutral conditions to the solutes. The acid soluble fraction of Fe was almost unavailable in the upper section or might have been leached to lower sections.

Manganese average concentrations followed the phase ordering: residual > reducible > acid soluble > oxidizable (Figure 5a,b) for both the cores. The percent contribution of Mn from all four fractions were 61, 20, 17 and 2 of residual, reducible, acid soluble and oxidizable respectively for Rigni Chhapra, however, it was 53, 26, 17, and 4 for residual, reducible, acid soluble and oxidizable for Chaube Chhapra. Potentially leachable fractions (acid soluble and reducible) differed with the depth in both the core profiles. Reducible fraction dominated in the upper ground while acid soluble fraction dominated in the lower ground. It had an association with the higher concentration of Fe and Mn in the upper ground which decreased with the depth in both the core profiles (Figure 4). Speciation study in the sediments of Nashina Lake (Heilongjiang, China) reported that Mn was typically found in Acid-extractable species or Fe-Mn oxide species, and thus can be easily remobilized and enter the food chain [62]. Residual phase, also known as the geochemical phase comprises naturally occurring minerals which may hold elements embed their crystalline matrix [63,64]. Weathering and mineralogy are the controlling factors of the elemental concentration in crystalline fractions which is not soluble under experimental conditions. The most abundant available fraction was observed reducible followed by an acid soluble in both the cores. Presence of a significant concentration of Mn in acid soluble or easily exchangeable form indicated that the element exists in the reducible form [4]. However, the oxidizable phase contributed only 2% and 4% respectively at Rigni Chhapra and Chaube Chhapra respectively due to rare or absence of sulfide minerals in both the cores [4].

Zinc in different extracted fractions followed a trend in both the cores, in the order-residual > acid soluble > reducible > oxidizable (Figure 5a,b). Residual phase was the most dominant phase with 65% and 79% at Rigni Chhapra and Chaube Chhapra respectively due to which the element bounds with mineral lattice and cannot be remobilized under natural conditions [64]. Acid soluble was the second most abundant in the fractions (19% and 10% respectively in core profiles of Rigni Chhapra and Chaube Chhapra) which bounds with carbonate due to the quite high stability of ZnCO_3_ under the Eh-pH settings of subsurface. It also has the character to get co-precipitated with CaCO_3_. Reducible fraction is the third most available fraction. This fraction represents the portion of elements bound to Fe and Mn oxides. It gets released to the soluble form when the matrix is subjected to reducing conditions. Oxidizable phase is the least abundant, only 3% and 2% at Rigni Chhapra and Chaube Chhapra, respectively. It shows that Zn is not found to be associated with organics and sulfides.

### 3.5. Risk Assessment Code (RAC)

**Figure 6:**
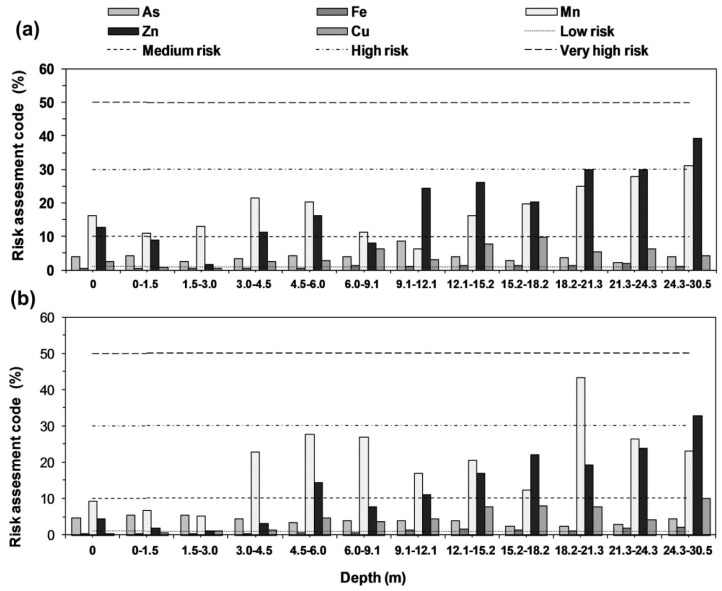
Calculated risk assessment code for As, Fe, Mn, Zn, and Cu (**a**) Rigni Chharpa (**b**) Chaube Chhapra.

Table S3 represents the risk assessment code (RAC) indicating the sediment (%) representing as exchangeable and carbonate fractions or soluble acid fraction. It can be categorized into safe for the environment (<1%) to highly dangerous (>50%) of the total elemental concentration in the same fraction. Higher the percentage of the element, more are the chances for it to enter into the food chain. It is useful to study to know the level of risk in existing subsurface environment. This code was applied to the results of the study and represented in (Figure 6a,b) for Rigni Chhapra and Chhapra respectively. For As in Rigni Chhapra RAC ranged from 2 to 8.7% while in Chaube Chhapra it ranged from 2.1 to 5.3% indicating risk (but low) at both the locations for its mobilization in the solute. In case of Fe and Cu, it was below low risk (range 0.8−1.7% for Fe and 0.5−9.7% for Cu), but it reached to medium risk zone for Mn (range 6.2−30.9%) and Zn (1.6−39.1%) in the core of Rigni Chhapra. The results were also same for Chaube Chhapra range for Fe (01−2%), Cu (range 0.1−10%), for Mn (5−43.3%) and Zn it ranged from 1 to 32.8% respectively. The reason could be the formation of carbonates complexes by Mn and Zn in the sediment formation. The values of RAC were lower in the upper unsaturated sections while it simultaneously increased with the vertical depth or below to water table.

### 3.6. Geo-Accumulation Index (I_geo_)

**Figure 7:**
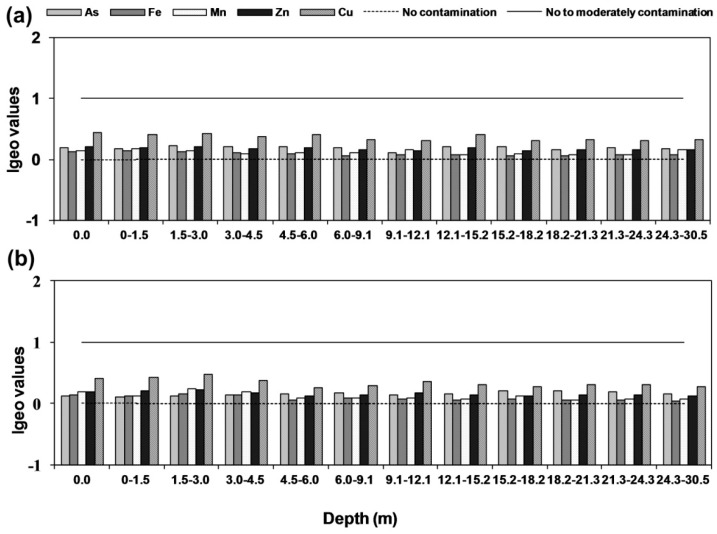
Calculated Igeo for As, Fe, Mn, Zn, and Cu (**a**) Rigni Chhapra (**b**) Chaube Chhapra.

The geo-accumulation value for all the five elements (As, Fe, Mn, Zn, Cu, and As) were calculated at every 1.5 m horizons in the vertical profile of both core sediments (Figure 7a,b). The results indicated that geo-accumulation index of both the core sediments fell under grade 0 and grade 1 which suggest uncontaminated as well as uncontaminated to moderately contaminated conditions, respectively. It was noticed that geo-accumulation for As fell in the range between uncontaminated to moderately contaminated except 3 and 9.1 mbgl in Rigni Chhapra. In the core of Chaube Chhapra, it was slightly different, where three depths 1.5, 3 and 12.2 m fell within uncontaminated while the rest fell in uncontaminated to the contaminated category. In case of other elements, i.e., Fe, Mn, Zn and Cu, the sediment of complete profile exhibited uncontaminated to contaminate category at both the locations.

### 3.7. Enrichment Factor (EF)

**Figure 8:**
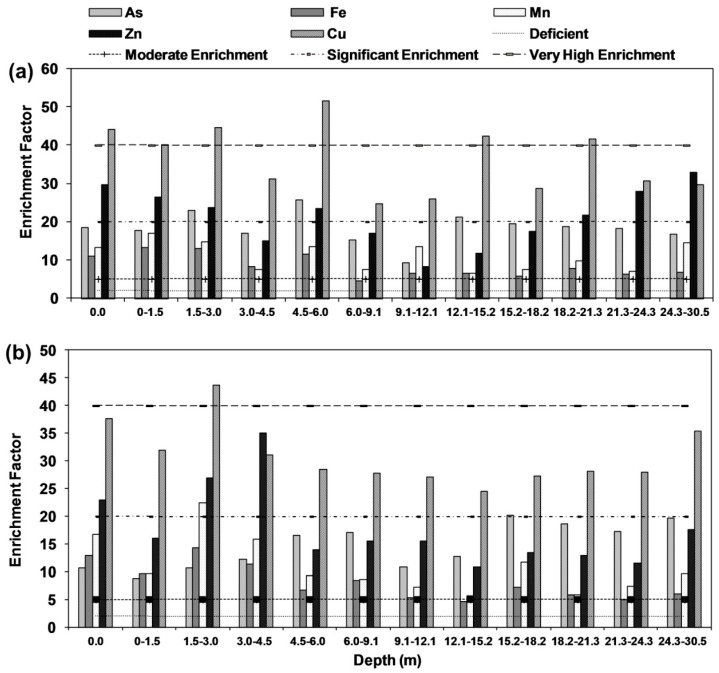
Calculated enrichment factor for As, Fe, Mn, Zn, and Cu (**a**) Rigni Chhapra (**b**) Chaube Chhapra.

An enrichment factor of the results was estimated and represented in (Figure 8a,b). It was observed that core sediments of Rigni Chhapra fell in significant enrichment except for depths of 1.5 and 6 m where it exceeded this limit. Iron remained in the moderate enrichment category except for upper section (top to 6.1 mbgl). Zinc fell in the significant enrichment category in whole profile in both the cores while Cu fell in very significant enrichment category in whole profile in both the cores.

### 3.8. Correlation Matrix

A Pearson correlation matrix among As and other associated parameters, i.e., Fe, Mn, Zn, Cu, organic carbon, carbonate, phosphate, sand, silt and clay was measured (Table 3). A positive and significant (*p* < 0.01) correlation of organic carbon with Fe (r^2^ = 0.86), and Cu (r^2^ = 0.78) was observed in Rigni Chhapra while a significant positive correlation of organic carbon with Fe (r^2^ = 0.88), Mn (r^2^ = 0.76), and Cu (r^2^ = 0.78) was observed in Chaube Chhapra. However, As did not correlate with organic carbon in the core of Rigni Chhapra while a significant negative correlation (r^2^ = −0.75) was observed in core sediments of Chaube Chhapra. The same trend was also observed for carbonate with Fe (r^2^ = 0.88), Mn (r^2^ = 0.88), Zn (r^2^ = 0.72) and Cu (r^2^ = 0.86), and a moderately negative correlation with As (r^2^ = −0.55; *p* < 0.05) in the core of Chaube Chhapra but it showed a positive correlation with Zn (r^2^ = 0.75) and a moderately significant with Mn (r^2^ = 0.44; *p* < 0.05) only. A weak and insignificant correlation of As with other elements (Fe, Mn, Zn, and Cu) was observed in Rigni Chhapra while negative but significant with Fe (r^2^ = −0.71), and Cu (−0.79) and a moderately significant negative correlation (*p* < 0.05) with Mn (r^2^ = −0.63), Zn (r^2^ = −0.57) in Chaube Chhapra. Phosphate did not exhibit any correlation with any analyzed elements. A significant correlation of Fe and Mn was observed with Zn and Cu. It suggests that these metals could be adsorbed onto the oxyhydroxides of Fe and Mn [65].

An interesting correlation of grain size of the core sediments was observed with analyzed elements. Sand content exhibited a significantly (*p* < 0.01) negative correlation with Fe (r^2^ = −95), Cu (r^2^ = −0.73) and moderately significant (*p* < 0.05) with Mn (r^2^ = −0.65) in core sediments of Rigni Chhapra. A significant (*p* < 0.01) but the negative correlation of sand was also observed with other elements Fe, Mn, Zn and Cu in core sediments of Chaube Chhapra. Correlation coefficient suggested that soil texture, especially clay and silt, plays a major role in elemental distribution in a agricultural subsurface sediments [60]. Arsenic did not exhibit any significant correlation with grain size in Rigni Chhapra, but it had a significant positive correlation with sand content (r^2^ = 0.72; *p* < 0.01) significant to moderately significant but negative with silt (r^2^ = −0.73) and clay (r^2^ = −65) respectively, in core sediments of Chaube Chhapra. The same results were observed in another study done in the Samastipur district of Bihar, India [8] where As showed a positive correlation with sand while the negative correlation with a silt content of sediments. Reference [66] documented that medium-textured sediments enriched with organic matter presumably resulted from the rapid accumulation of Holocene sediments are a key indicator of As risk areas. Organic carbon showed a significantly positive correlation with silt and clay fraction while it negatively correlated with sand in both the core sediments. Carbonate content also showed a significant positive correlation (*p* < 0.01) with silt and clay, and significantly negative correlation with a sand fraction in the core of Chaube Chhapra while no correlation in Rigni Chhapra.

### 3.9. Mineralogical Evidence of As and Other Elemental Occurrence and Compositions

**Figure 9:**
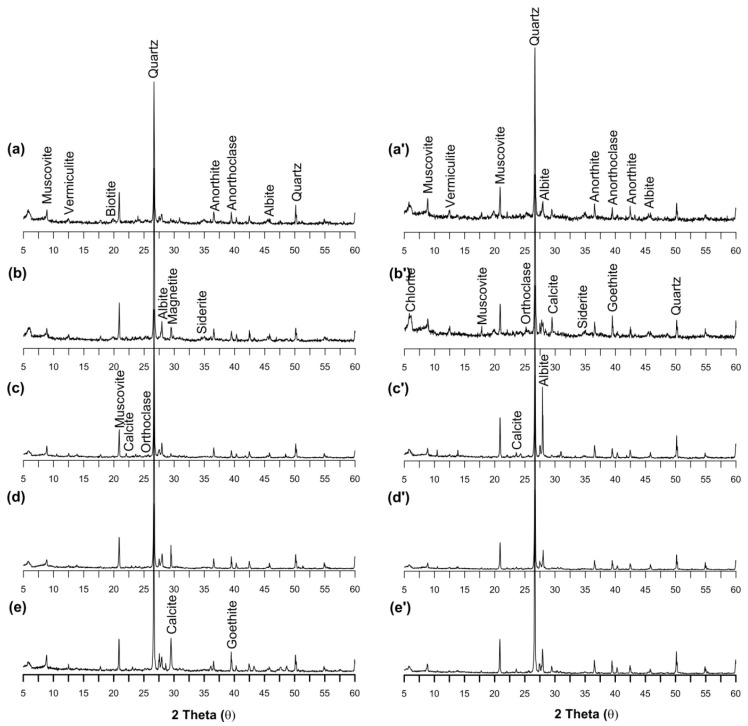
X-ray diffraction spectra for core profile of Rigni Chhapra with a comparision of five depths of (**a**) yellowish silty clay at 0.5 m (**b**) micaceous medium sand (dark grey) with OM at 1.5 m (**c**) yellowish grey fine sand at 6.1 m (**d**) grey to yellowish medium sand with OM at 15.2 m (**e**) grey fine sand with OM at 30.5 m and of core Chaube Chhapra (**a’**) yellowish silty clay at 1.5 m (**b’**) micaceous medium sand (dark grey) with OM at 3.0 m (**c’**) yellowish grey fine sand at 6.1 m (**d’**) grey to yellowish medium sand with OM at 15.2 m (**e’**) grey fine sand with OM at 30.5 m.

An attempt was taken to understand the mineralogical evidence for As and other element (Cu, Mn, Fe and Zn) occurrence and mobilization in the sediments of core profiles. The XRD analysis revealed that principal minerals present in the aquifer sediments of Rigni Chhapra were muscovite, quartz, albite, calcite (CaCO_3_), anorthite and orthoclase less abundant minerals like vermiculite, biotite, siderite, goethite and magnetite traced (Figure 9a). The abundance of chert and chlorite indicated the sedimentary and meta-sedimentary origin of sediments [67]. The detection of calcite and muscovite in the upper oxidized sediments indicated an association with chelation of the elements in the zone of oxidation [43].

A significant concentration of As was reported in core sediments in both the cores (Figure 4a,b). Peaks of goethite (FeO(OH)) were identified throughout the core profile, however, magnetite (Fe_3_O_4_) was identified only in the upper ground comprising yellowish oxidized silty clay as shown in (Figure 2). An average Fe concentration of 19,713 and 21,183 mg/kg was reported for Rigni Chhapra and Chaube Chhapra respectively. The mineralogical evidence could not revealed the presence of pyrite but enabled to identify the peaks of siderite and magnetite in the core sediments in a section of micaceous medium sand (dark grey) with organic matter at 1.5 m. Siderite (a secondary mineral) was identified in an early study of As in Southeast Asia by Islam et al. [68]. A higher As concentration (184 mg/kg) has been noticed in siderite, which included quartz—so the real concentration in the siderite could be higher [69]. It proved that siderite could be a sink for As in fluvial and deltaic regions. The occurrence of muscovite, chlorite and Fe-OH coated quartz in core sediments could be an additional cause for a higher concentration of As in these sections at Rigni Chhapra. There was no difference in mineralogy of Chaube Chhapra and Rigni Chhapra, but a interesting association of As with siderite mineral was noticed. A high concentration of As was found in the middle section of the core where siderite was traced (Figure 9b).

## 4. Conclusions

The sand fraction dominated throughout the core profiles of both the places except in the upper ground. The elemental concentration of As, Zn and Cu exceeded their corresponding average crustal values of 13, 95 and 45 mg/kg respectively at various depths in both the core profiles. The concentration of Fe and Mn remained below the average crustal values. Residual was most dominating fraction of all the analyzed elements, while the reducible fraction was the second most dominant fraction for As, Fe, Mn, and Cu respectively for both the cores. It may have been released when aquifers were subjected to changing redox conditions. The acid soluble fraction was of most interest because it can quickly mobilize into the water system which formed the third most dominating fraction. Four color code of sediments was applicable in core sediments for total As concentration, and no clear association was observed with any fraction of all elements analyzed except residual for As. Although, the core sediment was observed enriched with As and other elements (Cu, Fe, Mn, and Zn) it fell under uncontaminated to moderately contaminate which may exhibit low risk in prevailing natural conditions. XRD traces indicated the presence of siderite and magnetite in the core sediments in a section of dark grey with micaceous medium sand with organic matter (black).

## Figures and Tables

**Table 1:** Grain size distribution (in %) in the core sediments of Rigni Chhapra and Chaube Chhapra vertical below ground level.

Depth (m)	Rigni Chhapra			Chaube Chhapra		
	Sand	Silt	Clay	Sand	Silt	Clay
0.0	21.71	65.13	13.16	7.33	64.00	28.67
1.5	16.67	54.00	29.33	22.67	56.67	20.67
3.0	7.33	64.00	28.67	2.65	56.29	41.06
4.5	66.67	28.00	5.33	13.33	66.00	20.67
6.0	84.00	15.33	0.67	82.67	14.67	2.67
9.1	93.42	5.92	0.66	78.81	15.23	5.96
12.1	88.08	4.64	7.28	86.67	6.67	6.67
15.2	95.45	3.25	1.30	96.67	2.67	0.67
18.2	93.20	0.67	6.14	81.33	10.67	8.00
21.3	95.14	2.66	2.20	91.33	4.67	4.00
24.4	96.00	2.67	1.33	85.33	2.67	12.00
30.5	80.00	3.33	16.67	97.33	1.33	1.33

**Table 2:** The concentration of As and other elements in (mg/kg) dry wt. extracted from core sediments vertical below at Rigni Chhapra and Chaube Chhapra.

Depth (m)	Rigni Chhapra					Chaube Chhapra				
	As	Fe	Mn	Zn	Cu	As	Fe	Mn	Zn	Cu
0.0	11.98	26,248	566	140.2	98.8	7.65	33,777	783	120.2	93.1
1.5	11.40	31,088	711	124.2	89.1	7.50	30,216	546	100.3	95.3
3.0	14.17	28,996	597	107.1	95.1	7.69	37,052	1052	140.9	107.9
4.5	12.76	22,617	370	82.0	81.1	9.50	31,819	804	198.0	82.8
6.0	13.10	21,173	453	87.3	90.5	10.11	14,909	370	62.1	60.1
9.1	12.47	13,871	401	101.6	69.4	11.45	20,354	381	76.5	64.7
12.1	6.94	17,854	659	45.0	67.6	9.21	16,706	401	97.1	79.7
15.2	12.92	14,756	267	52.2	89.6	10.38	13,731	298	64.3	68.9
18.2	13.02	13,849	329	85.7	66.0	13.21	17,079	504	64.6	61.7
21.3	9.39	14,040	319	79.2	72.1	13.05	14,740	267	66.7	68.5
24.3	11.88	15,017	298	132.2	68.9	12.22	12,878	339	59.8	68.3
35.5	11.41	17,051	649	163.8	69.7	9.94	10,936	319	65.1	61.9

**Table 3:** Correlation among As and other parameters (**a**) Rigni Chhapra (**b**) Chaube Chhapra.

Variable	Fe	Mn	Zn	Cu	As	OC	Carbonate	Phosphate	Sand	Silt	Clay
(**a**)											
Fe	1										
Mn	0.67 *	1									
Zn	0.31	0.37	1								
Cu	0.76 **	0.25	0.10	1							
As	0.23	−0.29	0.27	0.46	1						
OC	0.87 **	0.60 *	0.56	0.78 **	0.24	1					
Carbonate	0.23	0.44	0.75 **	0.09	0.11	0.43	1				
Phosphorous	0.09	−0.09	0.28	0.29	0.13	0.37	0.08	1			
Sand	−0.95 **	−0.65 *	−0.41	−0.73 **	−0.27	−0.91 **	−0.18	−0.15	1		
Silt	0.93 **	0.54	0.35	0.80 **	0.31	0.91 **	0.09	0.29	−0.98 **	1	
Clay	0.81 **	0.78 **	0.45	0.44	0.13	0.73 **	0.34	−0.21	−0.87 **	0.75 **	1
(**b**)											
Fe	1										
Mn	0.93 **	1									
Zn	0.82 **	0.82 **	1								
Cu	0.89 **	0.83 **	0.69 *	1							
As	−0.71 **	−0.63 *	−0.57	−0.79 **	1						
OC	0.88 **	0.76 **	0.59 *	0.78 **	−0.75 **	1					
Carbonate	0.88 **	0.88 **	0.72 **	0.86 **	−0.55	0.76 **	1				
Phosphate	0.18	−0.08	0.03	0.23	−0.17	0.16	−0.02	1			
Sand	−0.98 **	−0.92 **	−0.83 **	−0.88 **	0.72 **	−0.91 **	−0.89 **	−0.15	1		
Silt	0.97 **	0.88 **	0.84 **	0.82 **	−0.73 **	0.90 **	0.81 **	0.19	−0.99 **	1	
Clay	0.92 **	0.94 **	0.71 *	0.92 **	−0.65 *	0.82 **	0.96 **	0.04	−0.93 **	0.85 **	1

* Correlation is significant at the 0.05 level (2-tailed); ** Correlation is significant at the 0.01 level (2-tailed).

## Supplementary Materials

The following are available online at http://www.mdpi.com/1660-4601/15/2/183/s1, Table S1: Sequential Extraction Procedure: modified BCR for elements (As, Fe, Mn, Zn and Cu) fractionation [1], Table S2: Risk assessment code (RAC) [2], Table S3: Geo-accumulation index and gradation, Table S4: Sediment quality and enrichment factor, Table S5: Physical parameters of core sediments (a) Rigni Chhapra (b) Chaube Chhapra.
