# Supplementary material for: Vertical Geochemical Variations and Speciation Studies of As, Fe, Mn, Zn, and Cu in the Sediments of the Central Gangetic Basin: Sequential Extraction and Statistical Approach
Source: Int J Environ Res Public Health. 2018 Jan 23;15(2):183. doi: 10.3390/ijerph15020183 (PMC5858258; doi:10.3390/ijerph15020183)
Supplement: Supplementary file 1 [file ijerph-15-00183-s001.pdf]

Supplementary material

Table S1. Sequential Extraction Procedure: modified BCR for elements (As, Fe, Mn, Zn and Cu) fractionation [1].

Fraction	Chemical Agent Added to the Residue	Duration	Fraction Extracted
1	40 mL 0.11 M acetic acid (CH_3COOH)	16h shaking (22 ± 5°C)	Easily exchangeable fraction
2	40 mL 0.1 M $NH_2OH.HCl$ (pH~2 with HNO_{3conc})	16h shaking (22 ± 5°C)	Reducible fraction
3	10 mL 8.8 M H_2O_2 (> 30 % W/V), shaking for 1 h at room temperature, heat in water bath for 1 hour until reduce to near dryness (<1 mL), repeat the processes, add 50 mL Ammonium acetate (CH_3COONa) (pH~ 2 with HNO_{3conc})	-	Oxidizable
4	Transfer the residue in to Teflon bomb and added 4 mL HCl_{conc}, 2ml HNO_{3conc}, and 2 mL HF_{conc} mixture	Heated for 30 min at 100 °C in the oven	Residual fraction

Table S2. Risk assessment code (RAC) [2].

Criteria	RAC (%)
No risk	< 1
Low risk	1–10
Medium risk	11–30
High risk	31–50
Very high risk	>50

Table S3. Geo-accumulation index and gradation.

I_{geo}	Grade	Pollution Degree
< 0	0	Uncontaminated
0–1	1	Uncontaminated to moderately contaminated
1–2	2	Moderately contaminated
2–3	3	Moderately to strongly contaminated
3–4	4	strongly contaminated
4–5	5	Strongly to extremely contaminated
>5	6	Extremely contaminated

Table S4. Sediment quality and enrichment factor.

Description of Sediment Quality	Enrichment Factor
Deficiency to minimal enrichment	<2
Moderate enrichment	2–5
Significant enrichment	5–20
Very high enrichment	20–40
Extremely high enrichment	>40

Table S5. Physical parameters of core sediments (**a**) Rigni Chhapra (**b**) Chaube Chhapra.

Depth (m)	pH	OM (%)	% C (tot)	OC (%)	Carbonate (%)	N (%)	Al_2O_3	P	SiO_2
					a				
0.5	6.50	4.39	1.22	0.53	2.96	0.07	7520	291	37,780

1.5	6.49	3.55	1.17	0.23	4.30	0.10	7440	129	33,700
3.0	6.69	3.13	0.97	0.23	1.15	0.06	7140	169	37,680
4.6	6.94	1.81	0.57	0.075	2.17	0.04	8700	207	44,230
6.1	7.01	1.80	0.66	0.3	3.84	0.07	5890	209	45,450
9.1	7.64	0.69	0.45	0.1	0.80	0.24	9450	168	49,170
12.2	7.67	0.55	0.34	0.1	0.22	0.14	8690	166	43,360
15.2	7.34	1.01	0.31	0.075	1.12	0.18	7090	153	49,070
18.3	7.50	0.46	0.45	0.1	0.92	0.16	7720	191	50,950
21.3	7.55	1.32	0.61	0.15	1.20	0.14	5810	186	45,430
24.4	7.80	0.74	0.71	0.1	2.58	0.17	7530	189	45,760
30.5	7.69	2.22	2.50	0.015	8.13	0.04	7900	181	40,980
				b					
0.5	6.62	5.10	1.58	0.98	4.17	0.19	8330	243	40,220
1.5	6.72	3.48	0.96	0.45	3.59	0.11	10,000	341	42,690
3.0	6.80	3.46	1.27	0.38	5.63	0.09	8310	177	43,930
4.6	6.95	2.44	1.17	0.23	4.00	0.08	8970	213	40,270
6.1	7.50	1.60	0.39	0.08	1.07	0.06	7100	198	54,320
9.1	7.40	1.32	0.57	0.15	2.52	0.01	7800	252	52,110
12.2	7.68	0.12	0.36	0.08	1.79	0.02	9900	300	54,260
15.2	8.00	0.11	0.35	0.08	1.67	0.01	9420	177	52,800
18.3	7.95	2.74	0.37	0.15	2.10	0.02	7610	243	51,580
21.3	7.80	0.76	0.43	0.10	2.28	0.01	8170	239	50,170
24.4	8.02	0.71	7.66	0.08	3.09	0.01	8230	213	49,660
30.5	8.10	0.72	0.68	0.08	3.15	0.01	5880	181	50,300

Units for rest parameters (mg/kg)

1. Rauret, G.; López-Sánchez, J.; Sahuquillo, A.; Rubio, R.; Davidson, C.; Ure, A.; Quevauviller, P. Improvement of the BCR three step sequential extraction procedure prior to the certification of new sediment and soil reference materials. *J. Environ. Monit.***1999**, *1*, 57–61
2. Perin, G.; Craboledda, L.; Lucchese, M.; Cirillo, R.; Dotta, L.; Zanette, M.; Orio, A. Heavy metal speciation in the sediments of northern Adriatic Sea. A new approach for environmental toxicity determination. *Heavy Metals Environ.* **1985**, *2*, 454–456.
